# The time benefits of young adult home stayers in France and Italy: a new perspective on the transition to adulthood?

**DOI:** 10.1186/s41118-017-0021-7

**Published:** 2017-07-25

**Authors:** Letizia Mencarini, Ariane Pailhé, Anne Solaz, Maria Letizia Tanturri

**Affiliations:** 1 Dondena Centre for Research on Social Dynamics and Public Policy Bocconi University, Via Roentgen 1, 20136 Milan, Italy; 2 133 bd Davout, 75020 Paris, France; 3 Department of Statistical Sciences, University of Padova, Via Cesare Battisti 241, 35121 Padova, Italy

**Keywords:** Transition to adulthood, Time use, Domestic work, Gender roles, Intergenerational exchanges, Italy, France

## Abstract

This article analyses how two co-residing generations contribute to the housework workload in Italy and France during the early 2000s. It studies the intergenerational exchange of time between young adults and their parents by indirectly comparing the level of domestic comfort enjoyed by young people in the two closely neighbouring countries. A focus on the reasons for staying in the parental home provides an explanation for the tendency of young Italian adults to prolong their stay in the family nest. The results of time-use surveys suggest that young Italians (especially young men) may benefit more than their French counterparts in co-residing with their parents. Beyond the compositional or structural effects, they perform fewer domestic tasks than their French counterparts, a result that is related to different cultural practices.

## Introduction

Young adults today are leaving their parental home later than some decades ago, and there is considerable interest among both sociologists and demographers in understanding the factors that may delay this process, i.e. various constraints which may prevent young people from gaining their independence. In this paper, we reverse this perspective by looking for a rationale that could make a prolonged stay in the parental nest a more desirable option. In the socio-demographic and economic literature, less attention has been paid to the conditions of parent-adult child co-residence and to the factors that might make the choice of co-residence particularly attractive or advantageous for young adults. Several studies have focused exclusively on the economic advantages for co-resident young adults (Alessie et al. 2006; Blossfeld et al. 2005, Le Blanc and Wolff 2006), for instance, saving money, avoiding housing costs, receiving funds, having a more comfortable dwelling and being protected in case of need (e.g. unemployment). Yet, the research largely neglects the time benefits enjoyed by co-resident children: opportunities to save time for leisure or other activities (such as studying or working) and to receive attention and transfers of time from parents (in terms of domestic tasks, for instance), both of which may considerably increase young adults’ well-being in the parental home.

Our idea is that a central role in a young adult’s choice to stay in the nest may be played by a higher degree of well-being and domestic comfort in the parental home compared to other living arrangements (living single on their own, co-residing with peers or living with a partner). Domestic well-being for co-resident adult children may perhaps be linked to the amount of unpaid work performed at home by the two co-residing generations. In particular, we assume that young adults have greater well-being in the parental home when they perform few domestic duties and benefit more from transferring domestic tasks to their parents. Indeed, it is possible that the costs and benefits associated with domestic activities are not equally distributed across co-resident generations and between genders in different contexts. Therefore, we argue that young people who benefit the most in terms of parental time transfers at home may be those who delay the transition to adulthood the longest. Similarly, we expect that young people with a higher incremental domestic workload and a steeper reduction in time transfers after transition to adulthood will be less eager to leave the “gilded cage” (Cook and Furstenberg 2002) of the parental family.

The originality of our paper lies in our focus on the intergenerational exchange of time in Italy and France in the early 2000s. In particular, we estimate, from a gender perspective, the relative contribution to domestic activities of young people living in the parental home or elsewhere. In this way, we indirectly compare the level of domestic comfort enjoyed by young people in Italy and in France, which—all things being equal—can be an additional reason for staying in the nest. Italy and France are two of the few countries for which we have fit the data to examine this issue. The time-use surveys conducted in the early 2000s interviewed all members of the household. Furthermore, these two closely neighbouring countries represent two highly interesting cases for exploring this issue, as they show some similarities in the economic constraints that young people may have to face, though the economic constraints seem harsher in Italy than France partly due to welfare support being less developed. In both countries (see Appendix) during the early 2000s, the youth unemployment rate was high (15% in France, 22% in Italy), and fixed-term employment contracts were highly prevalent (around 17–18% of young employees in both countries held temporary jobs), such that a large share of young people was neither in employment nor in education, and they were at risk of poverty and social exclusion, more so in Italy than in France. Welfare support for young people was more developed in France than in Italy (Appendix), where the familialist Mediterranean model continues to prevail and help from the state remains scarce (Thévenon, 2015). For instance, 35% of students in France receive grants, whereas they are only 8% in Italy, which is still the case in 2015. Housing subsidies for young adults are more developed in France and provide an incentive to parents and children to have independent dwellings (Laferrere and Le Blanc 2004). Except for these housing subsidies, social transfers dedicated to young people remain scarce in France (Thévenon, 2015).

Though young people in both countries still differ by their levels of religious attendance,^1^ they share many other comparable cultural features (see Appendix). They have similar participation rates in leisure activities such as cinema or sport, in social activities (weekly contacts with friends), and they had equal access to the internet in 2006. Moreover, the quality of domestic life is important in both Italy and in France (e.g. attention to meal preparation; see Davidson and Gauthier 2010).

Italy and France differ substantially in terms of transition to adulthood, as they each pertain to two different behavioural models (Iacovou 2002; Cavalli et al. 2008): France fits into the northern European model characterised by early home-leaving and multiple transitions on the path to marriage and parenthood, whereas Italy is in the “latest-late” (Billari and Liefbroer 2007) group, with late home-leaving and more direct transitions from the family of origin to life with a partner and parenthood. Italian young adults stay longer in the parental home than their French counterparts, though they are less likely to be tertiary educated. The median age at first leaving the parental home in France was 24.6 years for men and 22.7 for women in 2004, against 30.8 for men and 28.3 for women in Italy (Eurostat). 32.3% of French and 73.2% of Italian young people aged 20–29 were living with their parents in 2004. However, with the worsening of economic conditions from 2008, this share has increased in both countries. In addition to harsher economic conditions, one possible additional explanation for the persistently later departure of young Italian adults could be that they (especially men) enjoy greater comfort while living at home with their parents (a gilded cage) than their French peers.

In this paper, we use available time-use data for Italy and France in the early 2000s to describe three indicators of domestic comfort. We estimate the amount of time that young adults spend on domestic tasks when they live: (1) with their parents and (2) as singles or couples outside the parental home. In addition, we also try to assess (3) the transfer of time from parents (in terms of domestic services) enjoyed by young co-residing adults.

### Theoretical background and research hypotheses

There is an abundant literature devoted to the determinants of transition to adulthood and to cross-country differences in age at leaving the parental home in Europe (e.g. Billari et al. 2001; Corijn and Klijzing 2001). Some studies focus on different structural and economic constraints, while others emphasise cultural dissimilarities. However, less attention has been paid to the conditions of parent-adult child co-residence. The research focus is usually on the home “leavers” and not on the complementary population, i.e. the home “stayers”. Obviously, the reverse explanations proposed in the transition to adulthood literature for leaving the parental home are valid for those who stay, and the factors cited cannot be neglected, such as labour market constraints, i.e. job insecurity, unemployment and low income (Aassve et al. 2002); the housing market, i.e. difficult rental market and mortgages combined with high transaction costs (Alessie et al. 2004; Mencarini and Tanturri 2006; Tanturri 2016); and social norms (Billari and Liefbroer 2007). Yet, young adults can voluntarily decide to stay longer at home, for instance, to take advantage of the general material and psychological comfort of living with parents. Higher well-being of young people in the parental home could be a rationale for staying in the parental nest. Co-residence between young adults and their parents can also bring mutual gain for both generations (Cook and Furstenberg 2002). Despite the intuitive importance of such aspects, most studies consider not only the intergenerational exchanges to be marginal, but also the characteristics and behaviour of the parents; thus, they usually focus only on young people’s characteristics to explain their presence in the parental home. Moreover, when intergenerational transfers are also taken into account, it is mainly monetary exchanges that are generally considered (Molina 2004), though some recent studies have also taken into consideration the intergenerational transmission of time-use behaviour (Alvarez and Miles-Touya 2012; Solaz and Wolff 2015). A recently growing research on domestic time-use emphasises its economic contribution to national production (National Time Transfer Accounts project). In a comparative life-cycle approach, Zagheni and Zannella (2013) show that gender inequalities in time-use begin during the period of transition to adulthood.

The literature identifies many indicators to evaluate the level of comfort in the parental home: the quality of the relationship with parents, the degree of personal freedom (Cavalli et al. 2008; Rampazi 2008; Cook and Furstenberg 2002) and the material comfort of the dwelling (e.g. separate bedroom, possession of a car and so on). Some studies have directly estimated the subjective well-being of young adults living in the parental home. Two papers based on data from the World Value Survey produce inconsistent results: Manacorda and Moretti (2006) find a negative association in France between child happiness and co-residence in the parental home, and a positive one in Italy. Conversely, Billari and Tabellini (2011) use the same database to show that this association is never significant.^2^ Use of time is also an important aspect of everyday life that contributes to individuals’ well-being (Zabriskie and McCormick 2003). A relatively unexplored area in the literature is how domestic well-being in the parental home is linked to the amount of unpaid work performed at home by the two co-residing generations, which, in turn, is closely connected to their time use.

Some studies focus on the household input of children (Bianchi and Robinson 1997; Hofferth and Sandberg 2001) or teenagers (Benim and Edwards 1990; Bonke 2010; Price et al. 2009). The underlying idea is that household chores may compete with time devoted to studying or to developing cognitive skills (Zill, Nord and Loomis 1995), as occurs with any kind of work; thus, chores influence children’s current and future well-being. These studies show that teens spend a significant amount of time doing household work and that the demand for that work increases—particularly for girls—when mothers are employed, which supports the time availability hypothesis (Benim and Edwards 1990; Blair 1992). The family’s housing and living standards and the number of substitutes who perform housework are also factors that affect the amount of time children spend on housework (Bonke 2010).

The housework contribution of young adults living in the parental home has received less attention. However, when applied to young adults in the parental home, “the time availability hypothesis” would suggest that time spent by young adults on housework is higher when the mothers are working. From this we, derive our first hypothesis:

H1: Young adults whose mothers are employed contribute more to total household domestic tasks in both France and Italy.

Thus, in countries where female labour market participation is higher—France, in our case—a higher average participation is expected from young adults at home.

There is little overlap between time-use research and studies on transition to adulthood, even though—as underlined in a comparative study from a time-use perspective on transition to adulthood by Gauthier and Furstenberg (2002)—the transition to adulthood is reflected by major shifts in young people’s patterns of time use. Looking at the time spent on housework by young people aged 18–34 (living in the parental home or not), they find that, for women, time on housework increases with the transition to partnership and significantly so with the transition to parenthood, but it remains more constant for men (with only a small increase associated with transition to parenthood). They noted that transitions (from school to work, to partnership and to parenthood) involve very similar time reallocation in different countries, a remarkable finding, given the large cross-national differences in the timing of transition to adulthood. However, the study by Gauthier and Furstenberg (2002) does not make any distinction between different living arrangements (living in the parental family or elsewhere) before entering a union. By contrast, Anxo et al. (2011) show that time spent on unpaid work differs between singles living with their parents and those living on their own. It also shows that gender differences are perceptible throughout the life course, including at the early stage. Specifically for young people in both living arrangements (in the parental home or elsewhere), gender gaps in time spent on unpaid work are substantially wider in Italy than in France (Anxo et al. 2011). This may be the result of a gender-differentiated socialisation process (Bonke 2010) in those countries and may affect not only the time use of young people, but also their perceptions of the net benefits of living with parents or not. Thus, leaving the nest implies an increase in the time spent on domestic activities. Based on the literature and on different patterns in the transition to adulthood in Italy and France, one can argue that Italian young adults stay longer in the parental home not only because they perform fewer domestic duties there, but also because leaving the nest would imply a larger increase in time spent on domestic work than it would in France. Thus, by comparing the time use of young people when they live in or outside of the parental home, we expect to find that:

H2: The marginal cost (in terms of domestic time) of leaving the parental home is higher in Italy than in France.

The first two hypotheses concern only the time use of young adults living in the parental home or elsewhere; but previous studies on the transition to adulthood suggest that the behaviour of the parents of young adults should also be examined (Cook and Furstenberg 2002). The decision whether to co-reside or to leave the parental home concerns two types of actors, i.e., the parents and the young people. According to Billari and Tabellini (2011), “the late transition to adulthood of young Italians is explained essentially by their preference to co-reside with parents or by their parents’ preference to co-reside with children, or both”. Other studies on the consequences for parents of an empty nest show that parents may be reluctant for their child to leave home. It is not very clear whether the cost of having a young adult at home is offset by the satisfaction of seeing him/her daily at this stage, since the effects are very country-specific. For instance, Italian parents seem to be more (negatively) affected by the empty nest syndrome than their French counterparts (Mazzuco 2006) and to show a certain preference for co-residence (Manacorda and Moretti 2006). According to this rationale, parents may influence children’s choices and either encourage or discourage their leaving. However, it is plausible to hypothesise that parents who prefer to co-reside with their children (as is apparently the case for Italians) are also more willing to provide domestic services for them at home (Cook and Furstenberg 2002). The fact that a higher percentage of Italian mothers are out of the labour market may make it easier for them to offer these services.

Our paper assumes that the decision—sometimes very constrained—to leave home is made by the young adult, but the parents’ behaviour may influence this decision. In this line of reasoning, we argue that offering young adults a greater level of domestic comfort by providing them with meals, laundry and many other free services may indirectly contribute to delaying their transition to adulthood.

Since the parental input of time devoted directly to young adult children cannot be easily identified, as evidenced in previous literature (Budig and Folbre 2004), we use an indirect measure, i.e. the incremental time cost for the parents of a co-resident young adult child (Craig and Bittman 2008). According to the literature on different parenting styles and obligations across countries, as well as that on time-use profiles across the lifecycle, we can argue that parents’ domestic workload differs according to the age and number of children, even if it is not easy to disentangle how much of the cost is endogenous and how much is not (Anxo et al. 2011; Tanturri 2012). Child cost is usually examined only for children below age 18, sometimes by parity, and it remains a mostly unexplored area when the children are grown. However, in a context where children continue to live with their parents until age 30 and beyond, it seems very useful to extend the analysis over a longer period by taking into account the cost of young adults at home. Therefore, it is interesting to analyse how the increase in time spent on domestic tasks when children are at home varies between the two countries and to verify the following third hypothesis:

H3: Italian young adults receive more transfers of domestic tasks than French young adults when living in their parents’ home and, consequently, the incremental cost of having a young adult at home is higher for Italian than for French parents.

A corollary of our research hypotheses derives from the significant gender gap in age at leaving the parental home, as observed in the two countries:

Since daughters leave the parental home earlier than sons, we also expect domestic well-being at the parental home to be greater for sons than for daughters.

### Data, variables and research strategy

Analysis of the time spent by young people on domestic tasks requires precise information on their time use. Time-use surveys represent a unique source of information on daily activities. Individuals report their time use during a period of 24 h by providing extremely detailed information on the activities performed during that day, based on a grid of 10-min time intervals. Aside from the diary, all the data sets contain rich sets of information on the background and socioeconomic situation of individuals and households. We used the most recent comparable time-use survey suited for the analysis. The French time-use survey was conducted in 1998–1999 by the French National Institute of Statistics, and it is the last survey to provide time-schedule information on both the parents and their children.^3^ 15,441 respondents (belonging to 8186 households) filled in the daily booklet. The Italian survey was conducted in 2003^4^ by the Italian National Institute of Statistics, and 57,773 respondents (belonging to 21,075 households) filled in the daily diary.

### Variable of interest

Our variable of interest is the daily domestic time calculated from the booklet. We used a wide definition of the domestic activities, because young adults might help other household members in different ways. It takes into account the standard domestic chores such as cleaning, cooking, dish washing, food shopping, and care activities of all types, i.e. childcare and adult care. We also included activities sometimes considered as semi-leisure, such as looking after pets or gardening and maintenance. Gager et al. (1999) showed that teenagers tend to spend a considerable amount of time looking after pets.

### Empirical strategy

Regarding empirical strategy, we used a specific methodology and different samples for each of our research questions.

In order to answer our first research question, i.e. whether young adults in the parental home contribute more to household domestic tasks if their mother is employed, we selected a sample composed of all single young adults aged 18–35 and living with their two parents or step-parents.^5^ The sample comprised 1161 young adults living with their parents in France (629 men and 532 women) and 5551 in Italy (2985 men and 2566 women). We compared the participation of young people in domestic tasks in the two countries. We used a pooled dataset^6^ and estimated a multivariate Tobit model^7^ on the amount of domestic time spent by young adults living at the parental home, focusing on the significance of the dummy variable for Italy. We started with a basic model controlling only for country and day of the week, and we then introduced, step by step, covariates related to individual and household characteristics.

To answer our second research question on whether or not the marginal cost of leaving the parental home is higher in Italy (in terms of domestic time), we used a larger sample. Since the cross-sectional nature of the data does not allow us to observe domestic participation for the same individuals before and after leaving the parental home, we compared the time-use of different young adults according to their family situations: single in parental home, single living alone, in a childless couple and in a couple with children. The sample comprised all young adults aged 18–35 in the above situations, totalling 3924 persons in France (1896 men and 2028 women) and 10,102 in Italy (4861 men and 5241 women). We estimated the incremental domestic time after leaving the parental home, with a Tobit regression on the amount of domestic time by country and on a pooled sample. The covariates of interest relate to the family situation.

Thirdly, we followed a methodology proposed by Craig and Bittman (2008) as an indirect measure to examine transfers of domestic tasks received by young adults when living at their parents’ home, i.e. the time cost to parents for having a young adult at home. Our analysis focused on a sub-sample of parental couples aged 40–65 (2258 French and 11,766 Italian). The domestic work for parents is the dependent variable in this analysis, and we estimated the increment of domestic work for parents with an adult child (or more children) at home with respect to childless or empty nest households (Craig and Bittman 2008).^8^ Special attention was paid to the size and sex composition of the young adult siblings. Since the percentage of null participation was relatively low (except for fathers, but we kept to the same method for comparison), we performed standard OLS regressions.

Since behaviour regarding participation in domestic tasks differs greatly by gender, we systematically ran separate models for men and women.

### Control covariates

Regarding individual characteristics, we controlled for age and age squared, educational level and activity status. As for household variables, we controlled for household composition (i.e. number of children under 18, number of young adults, sex composition of the young adult siblings—only brothers for men—only sisters for women), the mother’s employment status (whether she works or not), her level of education, some characteristics of the dwelling (number of rooms and presence of a garden), an indicator of town size and, finally, access to domestic services (paid domestic help).

## Descriptive findings from TUS data: young adults living with their parents in France and Italy

**Fig. 1:**
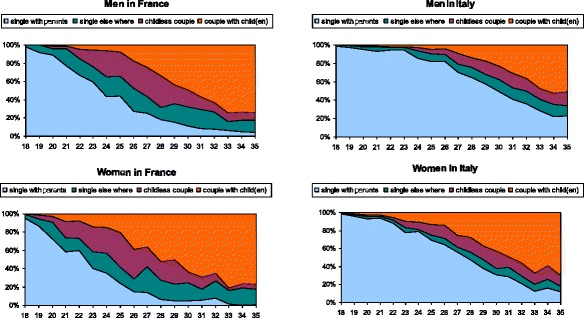
Living arrangements between 18–35 in France and Italy

According to the 2005 French and 2003 Italian Gender and Generation Surveys, more than 70% of men and over 53% of women in Italy aged 25–29 still live with their parents in their parental home, against less than 10% of their French peers. Intentions seem to drive behaviour: 80% of French men and 90% of French women aged 25–39 reported intending to leave the parental home within the next three years, versus 50% of Italian men and 70% of Italian women. The descriptive analysis of time-use data for France and Italy confirm this situation. Figure 1 shows the proportions of young women and men in France and in Italy according to their family situations and living arrangements, calculated from French and Italian time-use surveys. Italians are the least likely to have left home at all ages. The so-called “delay syndrome” (Livi Bacci 2001) affects all the stages of transition to adulthood. Living alone is a much less frequent outcome of leaving the parental nest in Italy. Partnership formation and the birth of first children also occur later in Italy than in France.

Differences in the timing of the transition out of the parental home also affect the composition of those living at home. Obviously, since Italian young adults leave home at a later age (Table 1), those still living with their parents are older on average than their French counterparts. They are also more likely to already have a job: 58 versus 25%. Half of French men living at home are still in education. Their family’s characteristics also differ. The most striking difference is that Italian young adults are less likely to have a working mother than French ones (less than 40% versus about 75% of French), and this characteristic can affect the time use of young people. Moreover, the observed differences between individuals living in the parental home in the two countries could also result from a different selection process of home-leaving. In this case, we would expect young adults staying at home in France to have been selected even more than those in Italy, where, conversely, prolonged co-residence is the norm.

**Table 1:** Description of young adults living with parents

	Men				Women			
	Italy		France		Italy		France	
Young adult characteristics	Mean	Std.	Mean	Std.	Mean	Std.	Mean	Std.
Age	24.64	*4.56*	21.97	*3.72*	23.75	*4.24*	21.22	*3.18*
Education								
High	0.08	*0.28*	0.16	*0.37*	0.10	*0.3*	0.20	*0.40*
Medium	0.49	*0.50*	0.28	*0.45*	0.61	*0.49*	0.33	*0.47*
Low	0.42	*0.49*	0.56	*0.50*	0.29	*0.45*	0.48	*0.50*
Professional situation								
Student	0.25	*0.43*	0.51	*0.50*	0.37	*0.48*	0.61	*0.49*
Unemployment OLF	0.17	*0.37*	0.14	*0.35*	0.22	*0.41*	0.14	*0.34*
Employed	0.58	*0.49*	0.35	*0.48*	0.41	*0.49*	0.25	*0.43*
Household characteristics								
Number of child(ren) <18	0.27	*0.55*	0.61	*0.94*	0.28	*0.56*	0.67	*0.97*
Number of young adults	1.90	*0.79*	1.58	*0.79*	1.96	*0.81*	1.71	*0.88*
Only sisters	0.62	*0.49*	0.61	*0.49*				
Only brothers					0.58	*0.49*	0.51	*0.50*
Mother’s work = yes	0.36	*0.48*	0.77	*0.42*	0.38	*0.48*	0.79	*0.41*
Mother’s education								
High	0.06	*0.23*	0.14	*0.35*	0.06	*0.24*	0.16	*0.37*
Medium	0.18	*0.39*	0.10	*0.30*	0.19	*0.39*	0.08	*0.27*
Low	0.76	*0.43*	0.76	*0.43*	0.75	*0.43*	0.76	*0.43*
Number of rooms	4.90	*1.61*	4.94	*1.17*	4.94	*1.68*	4.96	*1.16*
Garden	0.51	*0.5*	0.76	*0.43*	0.50	*0.50*	0.75	*0.43*
Paid domestic help	0.04	*0.19*	0.07	*0.25*	0.03	*0.18*	0.06	*0.23*
Sunday	0.31	*0.46*	0.15	*0.36*	0.32	*0.47*	0.13	*0.34*
Saturday	0.32	*0.47*	0.15	*0.35*	0.33	*0.47*	0.16	*0.37*
Week day	0.37	*0.48*	0.70	*0.46*	0.35	*0.48*	0.70	*0.46*
*N*	2985		629		2566		532	

Source: own calculations based on time-use surveys (Italy 2002–2003, France 1998–1999)

Standard errors in italics

Characteristics by sex and country. (mean and standard deviation)

## Results

### Young adults’ contribution to household domestic tasks

The time spent on domestic tasks by young men living in the parental home varies considerably between the two countries (Table 2). On average, Italian young men spend 35 min per day on these activities, which is substantially less time (−35%) than their French counterparts, who spend 54 min per day. Among young women, in contrast, there is not such a large difference, as Italian women spend roughly the same amount of time as French women on domestic tasks (respectively, 102 and 99 min per day). In fact, young women make a substantial contribution to household domestic tasks, representing nearly one fifth of the total domestic time (17% in Italy and 18% in France). Conversely, the male contribution is much lower in both countries. It is negligible in Italy, where young men assume only 5% of the total domestic workload of the household, but this is twice as high for young French men (10%).

**Table 2:** Domestic participation of young adults living at parental home in France and Italy by gender

	Men			Women		
	Italy	France	Sign.	Italy	France	Sign.
All						
Domestic time (min per day)	34.6 (69.7)	54.0 (87.3)	***	102.2 (107.9)	99.4 (107.1)	ns
Share of household domestic time (%)	5.4 (10.4)	10.3 (16.8)	***	16.5 (16.7)	17.6 (19.1)	ns
% of participants ^a^	42.5 (49.4)	54.1 (49.9)	***	79.5 (40.4)	78.6 (41.1)	ns
N	2985	629		2566	532	
Participants						
Domestic time (min per day)	81.5 (50.0)	99.9 (97.6)	***	128.6 (106.1)	126.6 (105.7)	ns
Share of household domestic time (%)	12.8 (9.3)	18.9 (18.8)	***	20.7 (16.2)	22.3 (18.9)	*
N	1268	340		2039	418	

Source: own calculations based on time-use surveys (Italy 2002–2003, France 1998–1999)

Standard errors in parentheses

*ns* no significance

Significance refers to the difference in the indicators between the two countries: *10%; **5%; ***1%

^a^Participants devote at least 10 min a day to domestic tasks

The low average time devoted by Italian young men to domestic labour results in part from the large share of young men who do no housework at all. Fewer than half (42%) of the young Italian men living in the parental home performed at least 10 min of domestic tasks on the interview day, while more than half did so in France (54%). Conversely, the proportion of young women taking part in domestic activities is much higher and is similar in both countries (around 79%). The time spent by young men who participate is sizeable (82 min for Italian young men and 100 min for French young men); but gender inequalities persist, since young women who take part in domestic labour spend about half an hour more than young men in France and around 50 min more in Italy.

To verify our first hypothesis that young people contribute more to total domestic household tasks when the mother works, we have to verify whether the country differences remain for men or reappear for women when we control for compositional effects (e.g. having a working mother). Indeed, once age, educational level, household composition, individual and mother’s activity status and dwelling characteristics are taken into account (variables introduced step by step), Italian young men still do fewer domestic tasks than their French counterparts (Table 3). Whatever the specification, the country coefficient is always significant for men. When all structural effects are controlled for, they spend 17 min less per day (marginal effect of Tobit model) on domestic duties in Italy than in France. Once all control variables are included, the country effect is also significantly negative for women, but weaker compared to men (the marginal effect is around 13 min). Thus, observable characteristics and structural effects do not explain all the differences observed between Italian and French young people, especially among men. Young Italian men with the same characteristics as their French counterparts spend less time on domestic tasks, and thus their well-being in the parental home may be greater. This greater domestic well-being is a potential explanation for their higher propensity to stay in the parental home. It is also true for women, but to a lesser extent. Therefore, we cannot reject our first hypothesis.

**Table 3:** Participation in domestic activities in minutes per day

	Only country variable	+ age	+ education	+ employment status	+ sibling size and composition	+ mother’s characteristics	+ home and domestic help
Men							
Italy (ref=France)	−45.754***	−53.310***	−52.004***	−50.280***	−48.854***	−43.570***	−37.918***
se	(6.588)	(6.761)	(6.892)	(6.909)	(7.152)	(7.523)	(7.705)
Marginal effect	−21.29	−25.22	−24.51	−23.58	−22.84	−20.1	−17.27
Women							
Italy (ref=France)	−2.254	−11.696*	−9.741	−17.556***	−16.608**	−14.282**	−15.185**
se	(6.481)	(6.609)	(6.765)	(6.523)	(6.723)	(7.033)	(7.317)
Marginal effect	−1.69	−8.9	−7.41	−13.65	−12.91	−11.09	−12.64

French (1999) and Italian (2003) time-use surveys, pooled data

Controlled always for interview day: + age, age squared, + education level, + activity status, + number of children under 18, number of young adults, sex composition of the young adults siblings, + mother’s employment status, mother’s level of education, + number of rooms, presence of a garden, access to domestic services, town size.

Standard errors in parentheses, *significant at 10%, **significant at 5%; ***significant at 1%

Tobit model comparing Italy with respect to France, with stepwise introduction of control variables

Analysing the changes in the value of the country dummy when the other covariates are added step by step into the model reveals how structural characteristics affect the difference in male and female participation in domestic duties between the two countries. The main changes occur when we control for age and mother’s characteristics as well as when home characteristics are added for men (Table 3). Controlling for age increases the negative country-specific effect, since young people living in the parental home are younger in France, and since younger adults spend less time on domestic activities than older ones. On the other hand, controlling for mother’s characteristics (her employment status and level of education) reduces the gap between countries. Hence, in France, young people’s mothers are more frequently in the labour market, and those with working mothers spend more time on domestic duties. Finally, wider use of paid domestic services in France (which reduces domestic time) also explains the decrease in the country dummy. For women, their employment status and the type of dwelling tend to increase the country-specificity of Italy.

Control covariates might have different effects in each country, however. For instance, as having a working mother is less frequent in Italy than in France, it might affect young adult participation differently because of selection effects. To explain more fully the determinants of young people’s domestic time in both countries, we estimated the same complete Tobit model (Table 4) separately on each country. Results show that Italian men increase their domestic participation as they get older, when they are highly educated or have more free time, i.e. when they are students or unemployed. Being unemployed has a positive effect on domestic time for French men. Employment status also plays an important role for both Italian and French women. Thus, it is evident that the domestic participation of young adults depends on the availability of time itself.

**Table 4:** Determinants of participation in domestic activities of young people living at parental home

	Men		Women	
	Italy	France	Italy	France
Age	22.603***	−17.317	5.89	8.401
	*6.935*	*18.738*	*6.768*	*20.962*
Age squared	−0.367***	0.421	−0.032	−0.062
	*0.133*	*0.373*	*0.133*	*0.437*
Education = high (ref = low)	23.046**	18.506	−14.168	−10.858
	*10.561*	*19.766*	*9.732*	*17.268*
Medium (ref = low)	2.461	16.213	−2.84	−26.778**
	*6.024*	*15.422*	*6.028*	*13.371*
Student (ref = employed)	26.393***	24.845	11.112*	18.397
	*7.883*	*17.788*	*6.324*	*16.746*
Unemployed/ OLF (ref = employed)	49.855***	74.081***	90.509***	99.769***
	*7.484*	*19.476*	*6.6*	*18.149*
Number of children <18	10.659*	−8.312	4.626	0.397
	*6.261*	*8.154*	*5.568*	*6.863*
Number of young adults	−5.528	−10.047	4.591	−12.167*
	*4.715*	*9.188*	*4.152*	*7.058*
Male siblings	2.163	−3.192		
	*6.678*	*15.602*		
Female siblings			−10.365*	−22.252*
			*5.86*	*13.414*
Mother in employment	11.246*	20.849	15.736***	10.567
	*5.945*	*16.57*	*5.333*	*14.378*
Mother’s education= medium (ref = low)	−9.887	12.13	−18.146***	−21.994
	*7.656*	*20.97*	*6.849*	*20.771*
Mother's education = high (ref = low)	−2.176	−20.272	−57.539***	−28.479*
	*12.568*	*20.624*	*11.473*	*17.058*
Sunday (ref=weekday)	−3.746	−5.355	−6.472	31.849**
	*6.608*	*17.948*	*5.965*	*16.006*
Saturday	40.857***	4.033	39.225***	48.094***
	*6.346*	*17.646*	*5.899*	*14.997*
Paid domestic help	7.593	6.193	17.416	1.646
	*14.903*	*27.066*	*14.531*	*25.52*
Big city (ref = rural)	−4.456	−20.533	−9.712	−25.552**
	*7.568*	*14.337*	*6.984*	*12.831*
Small city	−16.860***	−17.901	−7.512	−12.264
	*6.3*	*21.074*	*5.805*	*19.412*
Number of rooms	5.356***	5.895	0.478	−5.082
	*1.841*	*6.085*	*1.64*	*5.226*
Garden	9.637*	12.551	−3.491	8.739
	*5.696*	*16.052*	*5.294*	*14.264*
Constant	−408.601***	123.199	−65.773	−29.826
	*89.556*	*232.192*	*83.703*	*249.743*
Observations	2,965	595	2,539	500

French (1999) and Italian (2003) time-use surveys

Standard errors in italics

*Significant at 10%; **significant at 5%; ***significant at 1%

Estimates by gender and by country (Tobit model)

Beyond individual characteristics, youth participation is mainly driven by employment status and educational level of the mother. Having a working mother increases young Italian participation in domestic activities for both men and women. The stronger time constraints of working mothers favour their children’s participation in domestic duties. Moreover, growing up in a less traditional family—which marks a break with the male breadwinner model—apparently adheres to and transmits different norms about young people’s participation in domestic activities. Having a highly educated mother corresponds to a negative effect on the domestic participation of only young women in both France and Italy. This is because less time is spent on domestic tasks in such households, but also because a lower value is placed on such activities and this attitude is transmitted to daughters. Highly educated mothers may also seek to attenuate the gender division of roles by lessening their daughter’s participation in domestic chores. Finally, the time young women spend on domestic activities depends on the number of siblings having the same age. In particular, there are some economies of scale for young women who have only sisters. We can conclude that the participation of young adults in domestic tasks is essentially a “secondary” participation, strongly gendered and highly dependent both on their free time and on their mother’s participation in the labour market.

### The cost of leaving the parental home

To test our second hypothesis about the higher cost (in terms of domestic time) of leaving one’s family in Italy than in France, we calculate how much domestic labour young adults perform when they are outside the parental home, first separately by country and then by pooling the samples. In both countries, other things being equal, young people out of the parental home spend more time doing tasks than co-resident children (Table 5). Not surprisingly, this additional time is highest for young people living in a couple with children (about 104 min a day for men in both countries, compared to around 4 h for women), since it is well known that children are time-intensive (e.g. Anxo et al. 2011; Craig and Bittman 2008). However, the additional time is also remarkable for childless young people living in a couple as well as for singles. This result suggests that leaving the parental home implies additional housework for the young. In Italy, gender inequality increases among singles: single men spend 47 min more on domestic tasks than men living in the parental home, while the difference is 59 min for women. In France, marginal effects are quite similar: 58 min for men and 53 for women.

**Table 5:** Participation in domestic activities in minutes per day, by family situation for young people aged 18–35 (coefficients and marginal effects from a Tobit model)

	Men				Women			
	Italy		France		Italy		France	
	Coeff.	Marg. effect	Coeff.	Marg. effect	Coeff.	Marg. effect	Coeff.	Marg. effect
Single	74.21***	47.36	76.11***	57.78	61.12***	58.56	55.25***	52.73
	(8.25)	(5.96)	(14.76)	(11.93)	(10.07)	(9.79)	(15.29)	(14.77)
Childless couple	98.10***	64.86	78.73***	59.92	125.49***	121.31	93.84***	90.04
	(7.70)	(5.86)	(14.24)	(11.56)	(7.39)	(7.26)	(13.63)	(13.25)
Couple with child(ren)	156.65***	104.64	137.92***	102.53	297.02***	280.40	238.02***	222.01
	(6.50)	(4.94)	(13.17)	(10.17)	(6.18)	(5.73)	(12.75)	(11.58)
Observations	4861		1896		5241		2028	

Standard errors in parentheses, *significant at 10%, **significant at 5%, ***significant at 1%

Control covariates: age, age2, education, work status, number of rooms, garden, paid domestic help, town size, Saturday, Sunday

Reference: adult children in parental home

From the country effect estimates (using a pooled sample), we notice that whatever the family situation, Italian men spend less time on domestic work than their French counterparts, while Italian women spend more than French women (Table 6, Model A). The domestic time spent by young men living in the parental home is significantly^9^ lower in Italy than in France (Table 6 Model B). Similarly, being single, living in partnership or having children increases domestic time less strongly for men in Italy than in France. Therefore, Italian young men still continue to participate less in domestic tasks than their French counterparts after leaving the parental home. When they are single, on the one hand, they probably maintain strong family ties and pay frequent visits to parents who can help them with laundry, ironing, food preparation or other services; on the other hand, it is also possible that Italian single young men living alone reduce their domestic standards remarkably (e.g. cleanliness and meal preparation) compared to the parental home.^10^ For women, there is no country difference for young women living with their parents or for those living on their own. But Italian young women participate more than their French counterparts in domestic activities after couple formation. Entering a union lowers their domestic well-being by substantially increasing the domestic workload. This gap between Italian and French young women continues to increase with the birth of children.

**Table 6:** Participation in domestic activities in minutes per day by living arrangements and differences in participation between Italians and French (Tobit model)

	Men		Women	
	Model A	Model B	Model A	Model B
Italy	−41.52***	−53.99***	23.55***	−11.09
	(4.70)	(7.10)	(4.58)	(7.56)
Single	80.28***	75.95***	64.66***	37.73***
	(6.94)	(10.84)	(7.81)	(11.85)
Single* Italy		0.06		25.44*
		(12.70)		(14.53)
Childless couple	93.73***	75.98***	118.66***	81.21***
	(6.57)	(10.74)	(6.27)	(10.86)
Childless couple* Italy		−23.54*		43.94***
		(12.40)		(12.20)
Couple with child(ren)	151.46***	132.73***	283.43***	238.44***
	(5.82)	(9.20)	(5.52)	(9.30)
Couple with child(ren)* Italy		−25.08***		55.38***
		(9.54)		(9.26)
Differences: Italy minus France				
Young living with parents		−53.99***		−11.09
Singles		−53.93***		14.34
Couples		−77.53***		32.85***
Couples with child(ren)		−79.07***		44.29***
Observations	6757		7269	

French (1999) and Italian (2003) time-use surveys, pooled data

Controlled always for interview day: age, age squared, education level, activity status, number of rooms, presence of a garden, access to domestic services, town size, day of interview.

Standard errors in parentheses, *significant at 10%, **significant at 5%, ***significant at 1%

To summarise, leaving the nest implies a huge increase in domestic time. The marginal cost of leaving the parental home is lower for Italian young men than for French men, a result which does not seem to corroborate our second hypothesis, as it does not help to justify the longer delay of the Italians with respect to the French. However, it is difficult to say to what extent young people are able to anticipate the marginal cost of leaving the nest (in terms of domestic time). They might also place greater value on other factors of leaving the parental home—such as autonomy—while the level of participation in domestic tasks may not weigh heavily in such a decision. Lastly, this result could also be due to a selection process of the groups, which may differ by country. People who have already left home may be different from those who are still in the parental home.^11^

### The time benefits of living at home (i.e. the time cost for parents of young adults living at home)

To verify our third hypothesis, i.e. that Italian young adults receive larger transfers of domestic tasks than French young adults when living in their parents’ home, we calculate the additional time that parents spend on domestic tasks when adult children are present, compared to childless or empty nest couples with the same characteristics.

Ceteris paribus, having young adult children at home increases the domestic workload for the parents. This is always the case in Italy, usually to a greater extent, while in France this is the case only when there are two or three children and at least one is adult, or when there are at least three adult children (Table 7). In both countries, having adult children at home mainly affects mothers’ time use, but in most cases the cost is higher for the Italian mothers than for their French peers. Conversely, the effect of having adult children at home is never significant for fathers, except when they also have one younger child in France, and when there are three children in Italy—but in this latter case the effect is negative. This last result suggests that adult children may take their father’s place in performing domestic tasks. Italian fathers increase their participation only when they have younger children.

**Table 7:** Family composition effects on parents’ participation in domestic activities in minutes per day (OLS model)

	Italy			France		
Ref = no child no young adult	Couple	Mother	Father	Couple	Mother	Father
1 young adult	23.82***	28.79***	−4,97	21,75	20.72**	1,03
	(5.81)	(4.14)	(3.49)	(14.56)	(9.54)	(9.47)
1child <18	66.36***	51.87***	14.49***	65.63***	35.86***	29.77**
	(9.01)	(6.42)	(5.41)	(18.88)	(12.36)	(12.28)
2 young adults	45.00***	43.44***	1,56	19,22	13,4	5,81
	(6.54)	(4.66)	(3.92)	(20.48)	(13.41)	(13.32)
2 = 1 child + 1 young adult	66.17***	59.38***	6,79	71.36***	44.78***	26.58**
	(8.16)	(5.81)	(4.90)	(20.62)	(13.50)	(13.41)
2 children	119.68***	88.28***	31.40***	84.82***	78.34***	6,48
	(9.06)	(6.46)	(5.44)	(22.33)	(14.62)	(14.52)
≥3 young adults	38.79***	55.43***	−16.64**	97.17**	87.42***	9,74
	(13.97)	(9.95)	(8.38)	(44.69)	(29.26)	(29.06)
≥3 at least 1 child 1 young adult	73.10***	79.96***	−6,86	89.86***	83.33***	6,53
	(9.61)	(6.84)	(5.77)	(21.06)	(13.79)	13.70
≥3 children	152.82***	113.53***	39.29***	157.69***	127.20***	30,49
	(16.67)	(11.87)	(10.00)	(30.30)	(19.84)	(19.70)
Constant	318.32***	219.20***	99.13***	192.87***	135.97***	56,9
	(28.08)	(20.01)	(16.854)	(71.01)	(46.0)	(46.17)
Observations	11766	11766	11766	2258	2258	2258
*R*-squared	0,139	0,176	0,119	0,165	0,209	0,144

French (1999) and Italian (2003) time-use surveys

Controlled by women’s age, men’s age, couple employment status, couple educational level, number of rooms, presence of a garden, access to domestic services, town size, day of interview

*Significant at 10%, **significant at 5%, ***significant at 1%

In summary, we can conclude that Italian young people benefit from larger transfers of domestic work from their parents when they live in the parental home, but this is mainly thanks to their mother’s greater commitment. This result is confirmed even when we control for the professional situation of both parents. In this regard, it is useful to recall that in Italy, three young adults out of four have a non-working mother. Whereas in France, it is only when there are at least three adult children in the household that the young people benefit from larger transfers of domestic work, primarily from their mothers.

## Conclusions

Our study aims to understand how the norms and practices of domestic time exchanges between generations may contribute to explaining the delayed transition to adulthood in Italy. The comparison between Italy and France provides useful insights into the reasons behind differences in the timing of departure from the parental home. Our results suggest that young Italians may benefit more than their French counterparts from co-residing with their parents.

Time-use surveys are a valuable source for analysing in detail how unpaid work is shared among co-residing generations and between genders. The cross-sectional nature of the data does not allow dynamic analyses to verify the workload change for young people who leave the parental home; but, nevertheless, they provide interesting static comparisons between young men and women in different living arrangements.

Our results confirm that Italian young adults living with parents perform fewer domestic tasks than their French counterparts. Our findings prove that this result should not be considered as a purely compositional or structural effect, but rather as a truly different cultural practice. The low level of contribution is particularly evident for Italian men, who perform only 5% of total domestic production—half that of French men. Inter-country differences between young women are smaller while their contribution is more substantial (between 16 and 18%). These differences can therefore suggest that domestic well-being in the parental home may be greater for young men, especially for the Italians. We also determine a leaving cost for the young in terms of an increase in domestic time. This cost is equal for single women in both countries, greater for Italian women forming a partnership and lower for Italian men than for their French peers. The latter result invalidates our second hypothesis, however, Italian singles experience a larger decrease in domestic comfort than their French counterparts when they leave the nest, as they no longer benefit from the more generous services offered by their parents. Indeed, Italian parents, especially the mothers, bear a greater incremental cost when one young adult lives at home. Again, it is the Italian co-resident young adults who benefit from greater domestic well-being. The domestic well-being is always higher for young men than for young women in both countries. Women in the parental home contribute more than men to household tasks, confirming that gender role formation is a process which begins during childhood and is only amplified at the moment of couple formation and thereafter. Gender roles can be largely transmitted by parents, who themselves provide role models for children and have different expectations of boys’ and girls’ duties (Solaz and Wolff 2015), teaching them what a daughter and a son should do in accordance with the prevailing gendered social norms.

In summary, our results appear to confirm the idea that the parental family is a sort of “gilded cage” for young Italians, who perform only a small quantity of daily unpaid work and benefit from the care and attention of their parents (above all, mothers), which presumably makes their life at home very comfortable in this respect. Such a high quality domestic life (e.g. home-made meals and ironed clothes) is difficult to achieve when single or even in a couple. It is true that the increase in domestic work is lower for single Italian men than for their French peers, but if they do not replace the services offered by parents in the parental home with their own domestic work, they will experience a deterioration of well-being (for instance, frozen meals versus mamma’s cooking). Therefore, at least from this point of view, it can be perfectly rational for the Italians to postpone their departure from the family of origin and also to skip the step of living as a “single”. In France, the family demands more participation in domestic activities and provides fewer services, probably making co-residence less comfortable for young people and therefore reducing the incentive to remain in the nest.

Our results contribute to the debate on the factors driving the transition to adulthood by providing a new perspective on this issue. By focusing on the reasons for staying in the parental home and on intergenerational exchanges in terms of time, a further element is provided to explain the tendency of young Italian adults to prolong their stay in the family nest. Moreover, our results confirm that parents are important actors in the transition to adulthood, as their behaviour may actively influence their children’s degree of domestic well-being and make the parental home a particularly comfortable place to live.

Our study presents two major limitations linked to the data we have used. First, the time-use data are cross-sectional and do not allow us to follow young people in their process of leaving home. From a methodological point of view, this implies that we cannot apply a causal approach and therefore directly link the time use of young people (and the time transfers from their parents) with the choice to leave the family of origin. Second, in order to take advantage of comparable data between France and Italy, we have used data from the late 1990s and early 2000s, which might not represent the current conditions of the process of transition to adulthood, as this is now also severely influenced by the economic crisis (e.g. Giraldo and Mazzuco, 2016). However, recent data document that the delay of transition to adulthood has further increased with the economic crisis, but the distance between the proportion of young adults living in parental homes in France and Italy has remained almost unchanged.^12^

Despite these caveats, our analysis has clearly shown, on the one hand, that downward net intergenerational transfers of domestic time from the parents to the children make the life of Italian young male adults still living in the parental home exceptionally comfortable; and that, on the other hand, young women in both countries benefit much less from these time transfers. These two findings reveal strong gender differences among young adults co-residing with parents that mirror their parents’ behaviour. In a more comprehensive analysis of the factors delaying the transition to adulthood, these aspects of gendered intergenerational time transfer should be taken into account more, as most of the existing literature stresses only the importance of “monetary intergenerational transfers” from parents to children for the purposes of relieving their economic constraints.

## Appendix

**Table 8:** Indicators concerning youth conditions in France and Italy in the 2000s and 2010s

	France	Italy	France	Italy
Share of young people (20–29) living with their parents	2004		2013	
Men	39.5	79.8	43.2	83.4
Women	25.3	66.6	30.9	72.5
Estimated average age of leaving the parental household	2004		2015	
Women	22.7	28.3	23.0	29.0
Men	24.6	30.8	24.8	31.3
	2004		2015	
Share of young people (15–29) in education or training	52.5	59.0	43.0	50.6
	2000		2015	
Share of young people (15–29) neither in employment nor in education or training	13.1	19.6	14.7	25.7
Youth unemployment rate (20–29)	15.0	22.4	17.6	28.1
Youth employment rate (20–29)	40.9	32.6	40.5	24.4
	2004		2012	
Proportion of employees aged 25–29 in temporary jobs	18.3	17.2	23.2	28.3
Share of young people (20–29) at risk of poverty or social exclusion	2004		2013	
Living in parental home	21.6	27.3	21.6	32.4
Not living in parental home	23.1	32.4	25.5	35.6

Source: Eurostat, Youth database (http://ec.europa.eu/eurostat/web/youth/data/database)

**Table 9:** Indicators concerning welfare support for young people in France and Italy

	France	Italy
Upper age limit for child benefit that provides the family child allowances	19	17
Minimum age for benefit from social assistance	25	No age criteria
Median value of the transfers received by young people living in an independent household (2011)	2600	2400
Percentage of young people living outside the parental home and receiving a private transfer (2011)	18.54%	4.55%
Percentage of NEETs^a^ who do not receive any social transfers (average 2007–2011)		
Living with parents	20	48
Not living with parents	10	42
Share of students receiving grants (2015)	35	8
% of NEETs receiving housing benefits (2015)	42.90	3.50

Source: OECD, Social Policies for Youths, OECD Family database, European Commission

^a^NEETs = neither in employment nor in education or training

**Table 10:** Indicators concerning youth culture in France and Italy in 2006

	France	Italy
Frequency of never going to cinema (20–29)	24.0	25.1
Frequency of never going to or attending sport events (20–29)	61.4	58.5
Young people getting together with friends every week (20–29)	47.7	43.2
Participation of young people in church activities or other religious organisations (20–29)	0.4	15.4
Participation of young people in informal voluntary activities (20–29)	13.7	19.5
Daily Internet use (16–29)^a^	78.0	77.0

Source: Eurostat, Youth database (http://ec.europa.eu/eurostat/web/youth/data/database)

^a^This figure was available only in 2011

## Footnotes

1 15% of young Italian people participate to religious functions whereas less than 1% in France.

2 The authors suggest that the divergence may be due to the use of different samples in the two studies.

3 Only one or two persons per household were interviewed in the more recent survey, conducted in 2010, and not all of them were household members.

4 For comparison purpose, we selected the Italian survey closest to the French survey date.

5 We excluded one-parent households because it has been proven that a significant and complex association exists between family structure—in particular, that of children in non-intact families—and the nest-leaving of young adults. In a recent paper, Mencarini et al. (2012) used Generation and Gender Survey data for six European countries (Italy, France, Hungary, Bulgaria, Russia and Georgia) to show that children who have experienced parental separation tend to leave home earlier, but that the last child in the household—whose departure would thus leave the mother alone—tends to delay leaving.

6 This solution is feasible because time-use data were collected in a similar manner in the two surveys.

7 A Tobit model is estimated, since the distribution is left-censored, with many individuals for whom domestic time is null.

8 Since the data are cross-sectional, we could not run a causal analysis; our results nonetheless suggest that time spent on housework may be an important but underestimated factor in determining the home-leaving process.

9 To calculate interaction effects, consider the following equation: y=a*Italy+b*Single+c*(Single *Italy), with Italy and Single dummies, and Single*Italy the interaction term dummy. The parameter of French single (relative to reference category) is b, the parameter of Italian single is a+b+c. Similar behaviours between France and Italy are obtained by testing the following equality b=a+b+c, i.e. a+c=0 for each item of family situation covariate (Table 6).

10 In the datasets we use, there is no possibility of knowing such practical details about intergenerational ties.

11 Unfortunately, due to data limitations, we cannot go further to resolve this issue.

12 The proportion of 20–29-year-old young adults in the parental home in 2004 and 2013 remained double for men (and 2.5 times higher for women) in Italy compared to France (see Appendix).
